# Swedish Courts’ Evaluations of Interpreter-Mediated Child Investigative Interviews

**DOI:** 10.1177/10775595231162072

**Published:** 2023-03-07

**Authors:** Emelie Ernberg, Charlotte Löfgren, Linnea Koponen, Mikaela Magnusson

**Affiliations:** 1 Department of Psychology, 3570 University of Gothenburg, Sweden; 2 Barnafrid, Department of Biomedical and Clinical Sciences, Linköping University, Sweden

**Keywords:** child interviewing, child abuse, interpreting, language interpreters, legal decision making

## Abstract

Children can need the help of an interpreter if they are victims of a crime and need to be forensically interviewed in another language. Recent findings from practitioners raise concerns about the state of interpreter-mediated interviews with children. The current study aimed to explore how Swedish criminal courts reason when assessing interpreter-mediated and interpreter-absent (with children who are not fluent in Swedish) child investigative interviews. We conducted qualitative and descriptive analyses of written court verdicts involving 108 child victims who were evaluated to need an interpreter during their investigative interview. The courts frequently discussed issues regarding possible misinterpretations, language difficulties, and confusion. These perceived deficiencies in the interviews were often mentioned as a cause for assessing the child’s testimony with caution and, in some cases, as lowering the evidential value of the child interview. Possible implications for children’s legal rights are discussed.

The world is becoming increasingly global and multilingual, and Sweden is no exception. In 2021, approximately 20% of the Swedish population was born abroad (Central Bureau of Statistics, 2022). Further, it is estimated that about 30 percent of school-aged children in Sweden do not speak Swedish in their homes (Parkvall, 2019). Language interpreters can be necessary in the case that these children are victims of a crime and need to be interviewed by the police. During the last three decades, much research has been devoted to developing interview methods suitable to children’s developmental level and language skills (Newlin et al., 2015). Likely because of it being an emerging issue, there has been less research on interviewing children via an interpreter, although legal practitioners have reported concerns regarding the quality of interpreter-mediated child interviews (Ernberg et al., 2022; Fontes & Tishelman, 2016; Powell et al., 2017). To our knowledge, researchers have yet to examine if these concerns transfer over to legal decisions made regarding these children in court. The current study aimed to investigate if, and, in that case, how the presence or absence of an interpreter during investigative interviews with children who are not fluent in Swedish, are discussed in courts’ legal assessment of children’s testimony.

Non-Swedish speaking children living in Sweden can be especially vulnerable in child maltreatment contexts. There is no official data on the demographics of children interviewed via language interpreters. Between 2015 and 2020, 106,110 children applied for asylum in Sweden. The most common countries of origin for these children were Syria, Afghanistan, and Iraq (Swedish Migration Agency, 2022). Children have to flee their country of origin for numerous reasons, including armed conflicts, famine, and human right violations (Mattelin et al., 2022). Moreover, children in migration might have experienced adversities, including violent and traumatic events, both before and after fleeing their home country (Mattelin et al., 2021). Epidemiological research indicates that children in migration exhibit high prevalence rates of child maltreatment, including physical abuse (9–65%) and sexual abuse (5–20%; Jud et al., 2020). Furthermore, Swedish studies indicate that children with migration backgrounds report higher levels of child maltreatment compared to children without a migration background (e.g., Aho et al., 2016).

In previous studies on interpreter-mediated child investigative interviews, conducted in Sweden, Australia, and the United States, child interviewers have expressed several concerns regarding the current state of interpreter-mediated investigative interviews (Ernberg et al., 2022; Fontes & Tishelman, 2016; Powell et al., 2017). The interviewers mentioned problems of interpreters changing the format of questions (e.g., from open-ended to option posing) and the interview taking longer, which might exhaust the child. Further, many interviewers described a loss of control over the interview, and some interpreters were perceived as being uncomfortable speaking about sensitive topics such as sexual abuse, which might lead to children not getting the support they need to talk about their experiences (Lewy et al., 2015).

Sweden, alongside with the rest of the Nordic countries, have taken measures to ensure a child-friendly legal process for victimized children (see e.g., Johansson et al., 2017). As part of this model, children below 15 years of age typically do not appear in court (The Prosecution Authority, 2018). Instead, their video-recorded investigative interview from the preliminary investigation constitutes their legal testimony. Thus, the quality of the investigative interview, for example, the presence of leading questions, might not only affect the criminal investigation but also how the court assesses the credibility of a child witness or victim (Ernberg et al., 2020). As these cases often rely on little corroborative evidence, a credible testimony from a child is usually crucial for a conviction in cases involving child maltreatment (Ernberg et al., 2018a; Walsh et al., 2010). Investigating and prosecuting cases of alleged abuse against children is difficult for many reasons (see e.g., Cross et al., 2003; Ernberg et al., 2020). For example, children may be reluctant to disclose their abusive experiences, especially if the perpetrator is a family member (e.g., Magnusson et al., 2017).

The need for an interpreter during investigative interviews with children might cause additional challenges. While research in the area is scarce, the interpreter’s presence and the three-way communication that naturally ensues presumably further complicates and weighs on the child’s ability to disclose and speak about complex subjects. According to current guidelines by the Swedish Prosecution Authority (2018), child interviews should be conducted using authorized court interpreters if available. Authorized court interpreters have proved their knowledge of the Swedish legal system and relevant judicial terminology through written and oral examinations. However, practitioners report that there is a limited access to authorized court interpreters (Ernberg et al., 2022). Sweden had, in May 2022, 245 authorized court interpreters in total, and in several languages, for example, Dari and Vietnamese, there were no authorized court interpreters (The Legal, Financial, and Administrative Service Agency, 2022). These deficiencies might impact the interview quality if no interpreter with the required competency is available. If an interpreter is available but not in the vicinity, the interpretation might be conducted from a distance via telephone interpreting. Telephone interpreting has previously been found to have limitations, as interpreters have raised concerns about technology malfunctioning leading to, for example, difficulties in hearing (Wang, 2018a).

The current study aimed to explore how Swedish courts reason about the presence or absence of an interpreter and its effect on their decisions in cases when the child does not speak fluent Swedish, as well as their assessment of interpreters’ performance and children’s testimony. Due to the scarcity of research on this topic, we employed an exploratory approach using descriptives, exploratory quantitative analyses and qualitative analyses.

To study court reasoning, we analyzed written verdicts of cases with child complainants. Written verdicts are official documents containing information about the evidence presented and reasoning by the court in a specific case (Swedish Code of Judicial Procedure, Chap 30 par. 5). They are produced by the judge and published after deciding on a criminal case. Hence, written verdicts often include information about the court’s assessment of the child’s testimony (Ernberg et al., 2018b). Most court trials with child complainants are not open to the public, and other judicial documentation, such as preliminary case files, are restricted documents that are hard to get access to. Written verdicts were therefore the most usable source of data that was accessible for the current study. We searched verdicts from both district courts and courts of appeal, two of the three levels of general courts that deal with criminal cases in Sweden. The 48 district courts are the first instance for criminal cases, followed by the six courts of appeal in the country. The third level, the Supreme Court, mainly tries cases that could be important to the development of law (see The Ministry of Justice, 2015 for more information about the Swedish judicial system).

## Method

### Data collection

We collected written verdicts from Swedish district courts (*N* = 48) and courts of appeal (*N* = 6) from 2015 until 2021. To be included in the study, the verdict had to fulfill the following criteria: (1) the case contained a child complainant under 15 years of age, (2) the child was interviewed via an interpreter on at least one occasion due to the child not being fluent in Swedish; alternatively, the court mentioned the lack of an interpreter in their assessment of the child’s statement. The study was reviewed and approved by the Swedish Ethical Review Authority.

### Procedure

We used InfoTorg Juridik, a legal database containing verdicts from Swedish Courts, to collect written verdicts. An initial search was conducted in July 2021 using the search terms ‘the interpreter/interpreting’ and ‘child interview’ in Swedish, resulting in 79 verdicts of which 29 matched the inclusion criteria. A second, broader search was carried out in February 2022. All verdicts including the search phrase ‘interpret*' and ‘child interview*' (including words starting with these terms, such as “interpreter”, “interpreted” and “interpreting”) from January 1 2015 until December 31 2021 (*N* = 1090) were examined, and 43 additional verdicts that met our inclusion criteria were identified. The retrieved verdicts (*N* = 72) were then examined a second time, and 11 were omitted from the analyses due to, for example, children being interviewed as witnesses instead of complainants. Further searches were also conducted to identify additional verdicts where an interpreter should have been available at the child interview but was not appointed (e.g., verdicts where the court had difficulty assessing the child’s testimony due to language difficulties). Two searches, the first using the keywords ‘language difficulties' and ‘child interview’ (*N* = 58) and the second using the keywords ‘first language’ and ‘child interview’ (*N* = 41), resulted in 13 verdicts (8 and 4 respectively) of relevance for the study. Thus, the final sample consisted of 61 verdicts where the interviews had been interpreted and 13 verdicts where the court mentioned the lack of an interpreter (*N* = 74).

A secondary search was conducted in the legal database JUNO to identify cases that had been tried in a Court of Appeal. Due to time and resource constraints, we were unable to conduct a second full-scale search of all verdicts listed under the search phrase ‘interpret*' and ‘child interview’ (*N* = 555). Instead, each case number of the identified District court verdicts were manually searched using JUNO to identify appealed cases. Of the total sample of verdicts (*N* = 74), 26 (35%) was also tried in a Court of Appeal. None of the cases in this study had been tried in the Swedish Supreme Court. In some cases, the child had been victimized by more than one suspect, and some suspects had allegedly committed crimes against more than one child. Hence, in total, the data comprised cases from 74 verdicts, regarding 108 individual children, 122 unique child/perpetrator combinations, and 143 crime charges, representing 30 of Sweden’s 48 District Courts and five of the six Courts of Appeal.

### Coding manual

The coding manual used for the study was adopted from an earlier study by Ernberg et al. (2018b) and adapted for the present study. In verdicts with several child complainants, each child was coded separately. Some of the coded variables were the child and suspect’s age and gender, their relationship, and the assessed need for an interpreter. Further, we coded the court’s assessment of the child’s testimony, the interpreter’s performance, and whether the child’s statement was assessed with caution due to the interpreter’s presence or absence. All variables reported in the results section can be found in Appendix A, and the coding manual in full can be found at https://osf.io/mfvsg/?view_only=90659c9fcf974bfbada8ca65bb313791.

Two coders independently coded approximately 24% of the verdicts, with interrater reliability scores ranging between Cohen’s κ 0.84–1, indicating an adequate to perfect level of agreement across variables. Disagreements were solved by discussion. The first coder then conducted the remaining coding independently.

### Data Analysis

The verdicts were analyzed using both quantitative descriptive statistics and qualitative approaches. Content analysis was used to categorize the courts' considerations regarding the interpreted child interviews (e.g., Bengtsson, 2016). After reading the material thoroughly, the courts' statements were classified into codes closely resembling the original data, and codes that overlapped each other (e.g., “poor interpretation” and “interpretation has not worked”) were merged (“inadequate interpretation”) to create a coding structure. Next, two coders separately coded 41% of the qualitative data to assess inter-rater reliability. The total inter-rater agreement was .97, calculated by dividing instances of agreement by possible instances of agreement. The first coder then continued to code the remaining data independently. Since the purpose was to search for shared patterns affecting interpreted child interviews in court, only those statements mentioned in at least three cases are reported. Further, quotes to illustrate the courts' considerations were selected and translated to English by the first coder with minor adjustments to facilitate reading.

A series of chi-squared (𝜒2) tests were performed to explore possible relationships between several child- and interview-related factors and the case outcome. Since these analyses were exploratory, no hypotheses were stated.

## Results

### General Information About the Cases

General information about the cases is presented in Table 1. We aimed to code for the child’s native language and whether the child’s native language was used during the interpreted child interview, but information about these factors was very sparsely described in the verdicts and will therefore not be reported. Of the total sample of children (*N* = 108), 56 (51.9%) children were interviewed once, 30 (27.8%) twice, and 10 (9.3%) three times by the police. Information regarding the number of interviews was missing for 12 children (11.1%). All children were under the age of 15 and their testimony was therefore presented through their video-recorded police interview instead of directly in court.

**Table 1.** Case Characteristics.

Characteristics	Frequency (Proportion of valid observations)	Number of valid cases
Complainant gender		108
Girl	49 (45%)	
Boy	59 (55%)	
Complainant age		
At the onset of the alleged crime	*M* = 8.7 (*SD* = 3.1) range: 1 – 14 years	90
At the first investigative interview	*M* = 9.4 (*SD* = 2.9) range: 3 – 15 years	88
Defendant gender		82
Male	58 (69%)	
Female	26 (31%)	
Defendant relationship to complainant^a^		122
Biological parent	80 (66%)	
Stranger	15 (12%)	
Acquaintance	12 (10%)	
Step parent	8 (7%)	
Other person the child is dependent on, e.g.,a close relative or a babysitter	5 (4%)	
Sibling	2 (1%)	

^a^Based on 82 defendants, accused of committing crimes towards 108 complainants.

### The interpreted investigative interview

#### Presence of an interpreter

Crime characteristics, evidence and outcome in the cases are presented in Table 2. All child victims in the study (*N* = 108) were assessed by the police to require, or later by the court to have required, the assistance of an interpreter during the investigative interview. That is, in case the police had not appointed an interpreter for the child interview, the court discussed in the verdict that the child would have benefited from the assistance of an interpreter during the interview.

**Table 2.** Crime Characteristics, Evidence and Outcome.

Characteristics	Frequency (Proportion of valid observations)	Number of valid cases
Crime classification		143
Assault (including minor and aggravated assault)	77 (54%)	
Violation of children’s integrity (i.e., witnessing crimes between close relatives including domestic abuse)	19 (13%)	
Unlawful threat	15 (10%)	
Sexual offences, including (attempt to) rape and sexual assault	10 (7%)	
Other^a^	22 (16%)	
Repeated crime^b^		122
Yes	56 (46%)	
No	66 (54%)	
Evidence presented in court		122
Testimony from the complainant	122 (100%)	
Defendant admission of guilt (in full or partial)	44 (36%)	
Direct adult eyewitness	43 (32%)	
Evidence behavior^c^	66 (54%)	
Conclusive medical investigation	13 (11%)	
District court outcome		122
Convicted (on at least one charge against the child)	87 (71%)	
Acquitted (on all charges against the child)	35 (29%)	
Court of appeal outcome		40
Convicted (on at least one charge against the child)	31 (77%)	
Acquitted (on all charges against the child)	9 (23%)	
Penalty outcome		87
Prison	55 (63%)	
Probation	15 (17%)	
Fines or community service	13 (15%)	
Psychiatric care	4 (5%)	

^a^The remaining charges concerned harassment (n = 6, 4%), arson (n = 4, 3%), deprivation of liberty (n = 4, 3%), violation of a child’s integrity (n = 4, 3%), unlawful coercion (n = 2, 1.5%) and causing bodily injury (n = 2, 1.5%).

^b^The repeated crimes were ongoing from 1 to 56 months (M = 19.3, SD = 15.7).

^c^Testimony from, e.g., a parent, a therapist, a police officer, or a social secretary that the child exhibited distress after the alleged crime or that the child has shown a change in their personality or behavior following the alleged crime.

Of the child complainants included in the study, 81 (75%) were interviewed using an interpreter at least once during the investigation. For 17 (16%) children, an interpreter was not appointed, and 8 (7%) children were interviewed partially using an interpreter; for example, the interpreter was used as a backup if the investigative interviewer and the child had difficulties understanding each other. Information about to what extent an interpreter had been used was missing for two children, where the prosecutor had stated the need for an interpreter in the indictment.

Information regarding the interpreter mode (live or via telephone) during the investigative interview was only available for 27 children. Twenty-two children (20.4%) were interviewed using a telephone interpreter, and for five children (4.6%), the interpreter had been present during the interview. For the remaining children, interpretation mode during the child interview was not mentioned in the verdicts (*n* = 81, 75%).

#### Quality of the Interpretation

The quality of the interpretation was criticized for almost half (*n* = 38, 43%) of the children for whom an interpreter had been appointed in full or in part (*n* = 89). Specifically, concerns regarding the quality of interpretation were raised by the court (*n* = 30), another interpreter (*n* = 4), the defense (*n* = 3), or during the investigation (*n* = 1). For a few children, the interpreter’s performance was described as without remark (i.e., no issues to report; *n* = 7, 8%). The interpreter’s performance was not mentioned for the remaining children interviewed using an interpreter (*n* = 44, 49%).

Of the 38 children who were subject to interviews where the quality of the interpretation was criticized, one of the most common issues was perceived language difficulties between the interpreter and the child (*n* = 9, 24%), for example, suspected misinterpretations: “In general, in some instances, it has been difficult for the interpreters to understand what the children have said and - as the district court’s interpreters pointed out during the main hearing - there have been misinterpretations on several occasions.” (Verdict involving a 6-year-old and an 8-year-old victim)

Issues regarding phone interpretation were also brought up frequently, (*n* = 9, 24%), the most common problem with phone interpretation being that the interviewer had to repeat questions to the child due to the technique not working correctly (*n* = 4, 11%);

Since telephone interpretation has been used, questions have had to be repeated, and there have been difficulties hearing. During the interview, one of the interpreters stated that she had to interpret what she could hear, and it is evident that the interpretation is characterized by difficulties hearing, which invites error. (Verdict involving a 13-year-old, 12-year-old and two 8-year old victims)

Further, for a few children, courts mentioned that some words had been seemingly challenging to interpret (*n* = 4, 11%). Finally, in a few instances, the court just deemed the interpretation as inadequate (*n* = 8, 21%) or the interpretation being unreliable (*n* = 3, 8%) in short statements such as: “The interview was conducted using an interpreter, and the district court considers it evident that the interpretation did not work correctly.” (Verdict involving a 5-year-old child complainant)

### Courts’ Assessments of the Child Interviews

For 56 of the children, the presence (in partial or full; *n* = 39, 70%), or absence (*n* = 17, 30%), of an interpreter during the investigative interview was discussed by the court as affecting their assessment of the child’s testimony. When an interpreter’s presence during the child interview was discussed (*n* = 39), courts frequently mentioned that the child’s statement had to be evaluated with caution because the interview was interpreted (*n* = 19, 49%).

In this case, everyone involved has been interviewed in languages other than Swedish. It is not possible to in detail determine the quality of the interpretation. In general, however, it can be said that there is always a risk that details, and linguistic nuances are mistaken or distorted when interpreting. All in all, the above means that great care is required when the statements of those involved are to be evaluated. (Verdict involving a 5-year-old child complainant)

The most frequently mentioned reason for why a testimony should be evaluated with caution was that language difficulties (for example, the interpreter or the interviewer not understanding what the complainant was trying to explain) might have affected the child’s testimony (*n* = 8, 21%). In some cases, courts assessed the child’s testimony as less credible (*n* = 3, 8%) or even of lower evidential value due to the interpretation (*n* = 6, 15%); “The district court considers the child interview of low evidential value as it has emerged that the complainant’s statement during the interview has not been interpreted correctly by the hired interpreter.” (Verdict involving an 11-year-old complainant)

Further, for some children, the verdict just mentioned the interpretation being inadequate (*n* = 8, 21%) or unreliable (*n* = 3, 8%) and therefore negatively affecting the credibility of the child’s testimony: “A further circumstance that gives reason to assess the complainant’s information with caution is that the interpretation during the interview has obviously been deficient in certain parts.” (Verdict involving a 6-year-old complainant)

Finally, for almost half of the children interviewed using a telephone interpreter (*n* = 22), the interpretation mode was assessed to negatively affect the credibility of children’s statements (*n* = 9, 41%), often due to the interviewer having to repeat questions to the child (*n* = 4, 18%).

When an interpreter was not used during the interview (*n* = 17), the most frequent comments from the court when assessing the child’s testimony concerned possible language confusion or difficulties that might have occurred during the interview (*n* = 9, 53%), either because the interview had not been conducted in the child’s native language (*n* = 6, 35%) or since the child was assessed as not fluent enough in Swedish (*n* = 5, 29%). For example:

Both children are Turkish-speaking, and it is difficult to assess whether they understood the questions from the interviewer correctly. For the same reason, it is difficult to determine whether the answers given by the children may have been affected by language difficulties. This uncertainty about the language is a circumstance that further calls for caution in assessing the credibility of the information that emerged during the interviews. (Verdict involving a 8-year-old and a 7-year old complainant)

### Case Outcome

A series of chi-squared (𝜒2) tests were performed to explore possible relationships between several child- and interview-related factors and case outcome (conviction vs acquittal, see Table 3 for descriptive statistics). No expected cell frequencies were less than 5. There was no statistically significant relationship between child gender and outcome, 𝜒2 (1, *N* = 122) = 0.71 (*p* = .41), child age (preschool, school age, or adolescence, see Table 3) and outcome, 𝜒2 (2, *N* = 102) = 5.03 (*p* = .08), or interpreters’ presence or absence and outcome, 𝜒2 (1, *N* = 122) = 0.71 (*p* = .40). It was not possible to investigate the relationship between interpreting mode (telephone interpreting vs interpreting on site) and case outcome since information about interpreting mode was missing in many verdicts. Two final 𝜒2 tests explored possible relationships between the interpretation being criticized, the interpretation (or lack thereof) being assessed by the court as affecting the child’s testimony, and the case outcome. There was no significant relationship between the interpretation being merely criticized and the outcome of the case, 𝜒2 (1, *N* = 122) = 0.61 (*p* = .44). However, results indicate that if the court expressed concerns regarding the quality of interpretation or the lack of an interpreter as affecting their assessment of the child’s testimony, the defendant was less likely to be convicted, 𝜒2 (1, *N* = 122) = 6.77 (*p* = 0.009), rφ = 0.24.

**Table 3.** Case Outcome Depending on Interpreters’ Presence or Absence, Interpretation Mode, the Child’s Gender and Age at the First Interview.

	Frequency (Proportion of valid observations)		Number of valid cases^a^
	Conviction^b^	Acquittal	
Interpreter present	71 (74%)	25 (26%)	96
Telephone interpreter	14 (64%)	8 (36%)	22
Interpreter absent	16 (62%)	10 (38%)	26
Gender			122
Boy	52 (74%)	18 (26%)	70
Girl	35 (67%)	17 (33%)	52
Child age^c^			102^d^
Pre-school (3–6)	11 (61%)	7 (39%)	18
School-age (7–12)	42 (66%)	22 (34%)	64
Adolescent (13–15)	18 (90%)	2 (10%)	20
Total cases	87 (71%)	35 (29%)	122

^a^The table is based on the total number of cases since some alleged perpetrators were convicted of crimes towards one child but acquitted of others, and for some children one of their alleged perpetrators were convicted and another acquitted.

^b^Convicted on at least one count.

^c^At the time of the first child interview.

^d^Information about the child’s age at the first interview was missing in 20 cases.

## Discussion

The present study aimed to examine if practitioners’ concerns regarding the quality of interpreter-mediated investigative interviews with children translate to legal decisions regarding these children. To do so, we examined Swedish courts’ evaluations of interpreter-mediated and interpreter-absent investigative interviews with children who were not fluent in Swedish. Worrisomely, the results highlighted several problems that could compromise these children’s right to be heard. For almost half of the children who had been interviewed via an interpreter, the interpretation was criticized during trial. In line with concerns raised by Swedish investigative child interviewers (Ernberg et al., 2022), the courts frequently discussed perceived language difficulties and possible misinterpretations in their written statements. While assessing a child’s testimony is not an easy task during the best of circumstances, courts encounter particular difficulties assessing the content of testimonies given via interpreters in other languages. Accordingly, when an interpreter was used during the investigative interview, courts frequently expressed that the child’s statement had to be evaluated with caution. Further, results indicate that when the court did have reservations in their assessment of the interpreted child interview, the alleged perpetrator was less frequently convicted. Hence, the presence of language difficulties and other concerns regarding the quality of interpretation could sometimes negatively affect the evidential assessment of children’s testimony and possibly also the child’s legal rights.

The mode of interpretation was rarely mentioned in the verdicts and the information that was available primarily concerned telephone interpreting. Courts expressed concerns that interpreters could not hear the child properly since the technology worked poorly, and that interviewers needed to repeat their questions. The use of telephone interpretation could in some instances negatively affect the courts’ assessment of the child’s testimony. In a previous study by Wang (2018a), interpreters mentioned difficulties hearing as the main struggle with telephone interpreting, and one of the main coping strategies was repeating or asking for clarification. However, studies show that children sometimes change their responses when asked repeated questions, and these questions should therefore be avoided or carefully administered if needed (Krähenbühl et al., 2009). Interpreters also mentioned the difficulty with turn-taking when not being able to see the other parties in the conversation. Hence, telephone interpreting might come with increased risks when interviewing children about sensitive topics. Other problems regarding telephone interpreting have been raised by both police officers (Goodman-Delahunty & Mastschuk, 2016; Wakefield et al., 2015) and interpreters (Hale et al., 2022; Wang, 2018b), including poor sound quality, overlapping speech and confusion about the interpreter’s role. The accuracy of interpreting may decrease when the interpreter interprets via telephone (Määttä, 2018). However, in cases where it is not possible to have the interpreter in the room (e.g., due to difficulties finding a suitable interpreter in the area), remote interpreting might be the only option. One possible solution to the need of remote interpreting could be video interpreting (i.e., interpreting via video-conference technology). A recent study by Hale and colleagues (2022) found that interpreting performance (including accuracy) was lower in remote audio interpreting, compared to face-to-face interpreting, whereas interpreting performance in video interpreting did not differ from face-to-face interpreting. Using video interpreting in child forensic interviews would require improved, secure video-conferencing technologies for the police, and further research is needed to map the benefits and risks of video interpreting in child forensic interviews.

The courts discussed issues relating to language confusion and comprehension when children had been interviewed in Swedish (without an interpreter) despite having another first language. In addition, concerns were raised that the child may not have understood the interviewer or the questions correctly, which in turn might have affected the accuracy of their responses. The limited access to interpreters in certain languages might explain why some children were interviewed in their secondary language. Efforts clearly must be made to ensure that all children will have access to qualified interpreters when asked to give legal testimony (Ernberg et al., 2022; The Swedish National Courts Administration, 2017).

Interviewing witnesses in another language than that of the context of the event may also affect the witness’s memory retrieval of the event. According to the theory of encoding specificity and retrieval of episodic memories, details of episodic memories are more easily available when the context of retrieval is similar to the context of encoding the memory (Tulving & Thomson, 1973), and this has been seen to apply even for language context (Marian & Kaushanskaya, 2007). Thus, if the event happened in a context where the child’s first language was used, it might be beneficial to interview the child in the same language. Further research could examine this in a child interviewing context as this could be especially useful in cases where an interviewer who speaks the child’s language is readily available. It should however be noted that in certain jurisdictions, such as Sweden, it is required that everything said during a trial is translated into the official language (even if everyone present understands what is being said; The Courts of Sweden, 2021) meaning that such suggestions are not currently practically applicable everywhere.

The results from the present study are especially concerning given that children with a migration background might be especially vulnerable victims. Research indicates that these children are at a higher risk of experiencing childhood abuse and maltreatment compared to children without a migration background (e.g., Aho et al., 2016; Jud et al., 2020). Given that more than 24 000 cases of child physical abuse was reported in 2021 alone (BRÅ, 2022), the fact that we only managed to identify legal cases involving 108 children who needed the assistance of an interpreter during a 5-year period might indicate that many of these cases are not prosecuted. An interesting topic for future research would be to examine how cases involving children who are interviewed via an interpreter progress throughout the legal system. Moreover, children in migration might have experienced adversities, including violent and traumatic events, both before and after fleeing their home country (Mattelin et al., 2021). This further highlights the need for a well-functioning legal system that is adapted to the needs for these vulnerable children, and the current study indicates that the current state of the legal system is not up to par.

In 2020, the Convention on the Rights of the Child was incorporated into Swedish law (SFS 2018:1197). According to the Convention, children have a right to freely express their views and be heard in all matters concerning their lives, regardless of their gender, ethnicity, language, and other individual factors (UNCRC, 1989). Still, the present study adds to the growing literature that indicates that poorly conducted interpreter-mediated child interviews could potentially harm the child’s right to be heard during a criminal investigation (Ernberg et al., 2022; Fontes & Tishelman, 2016; Powell et al., 2017) and, further, have a negative effect on the assessment of the child’s statement in court. These results underline the importance of using qualified interpreters with competence for the task at hand. Specialized training initiatives focused on interpreting investigative interviews with children may provide a promising way forward (see Finnish Institute for Health and Welfare, 2020).

### Limitations

Before moving on to the conclusions, some methodological limitations need to be discussed. As is often the case with archival data, all information of interest was not accessible. Written verdicts do not follow a standard format in their reasoning and information regarding several variables was only available in so few verdicts that the value of coding them subsided. Hence, the results presented in the present study reflect the information given by the courts and might not correspond to the real complexity of the cases (e.g., differences in performance between authorized and non-authorized interpreters).

Furthermore, we coded verdicts that mentioned the use of, or lack of, an interpreter during the child interview. However, as courts are by no means obliged to mention the use of an interpreter in the verdicts, we have likely missed verdicts where an interpreter had been appointed to the investigative interview but was not mentioned in the verdict. Hence, the current results might not be representable for the majority of cases.

Finally, due to power issues, the results from the explorative analyses regarding possible relationships between a range of child- and interview-related factors and case outcome should be interpreted with caution. Although we made substantial efforts to identify all court cases during a 7-year period (2015–2021), the final sample size was relatively small (74 verdicts) and relevant information was missing from the verdicts.

## Conclusion

Given the lack of past research on this topic, the current study contributes important insights into court evaluations of interpreter-mediated child interviews in a forensic context. In many verdicts, courts discussed perceived issues regarding the interpreted-mediated or interpreted-absent child investigative interviews, such as misinterpretations, language confusion for children interviewed in their second language, and, in cases of telephone interpretation, problems hearing the child. Consequently, the child’s statement was evaluated with caution or assessed as having a lower evidential value. Thus, both the absence of an interpreter when needed and interpretation problems could compromise children’s right to be heard in legal cases that affect their life. Therefore, more effort should be focused on ensuring that all child witnesses and victims, despite their first language, are being treated fairly by the legal system.

## Appendix

**Table:**

	Variable	Variable explanation
1	Number coding for district courts	Which of Sweden’s 48 district courts handled the case
2	Defendant gender	0 = man 1 = woman
3	Number of plaintiffs under the age of 15	The number of plaintiffs under 15 years who have been exposed to crime in the same verdict (not by the same perpetrator on different occasions)
Each child plaintiff is coded on a separate row		
4	Child plaintiff gender	0 = boy 1 = girl
5	Child plaintiff’s native language	What is the child’s native language?
6	Child plaintiff’s age at the onset of the crime	The age of the plaintiff at the onset of the crime. Years are rounded down when we do not know the exact year
7	Child plaintiff’s age at the first investigative intervie	The age of the plaintiff at the first investigative interview. Years are rounded down when we do not know the exact year
8a	Plaintiff-defendant relationship	1. Biological parent
		2. Adoptive parent
		3. Stepparent
		4. Foster care parent
		5. Sibling
		6. Step/foster care sibling
		7. Another close relative
		8. Acquainted to the family
		9. Pre-school or school personnel
		10. Another person to whom the child is dependent
		11. Stranger
		12. Girlfriend/boyfriend
		13. Friend
		14. Acquainted to the child
8b	Relationship explanation	String variable with a clearer description of the relationship between parties, especially in cases with another person to whom the child is dependent
9	Does the child need an interpreter?	0 = No
		1 = yes, the court, the prosecutor, the police, or the child counsel assess that the child needs an interpreter during the child interview. Alternatively, the verdict or the actors mentioned above assess that the child would have needed an interpreter
10	Oral evidence	1. Video recorded forensic interview
		2. Video recorded interview (e.g., by a parent)
		3. Testifies in court
		4. Does not testify (e.g., very young children)
11a	Number of forensic interviews	The number of child forensic interviews described in the verdict
11b	How many forensic interviews have been held with an interpreter present	Describe the total number of interviews with the child using an interpreter
12	Was an interpreter used during the interview (code for each interpreted interview)	1. No
		2. Yes, a telephone interpreter
		3. Yes, a contact interpreter
		4. Yes, through a video link
		5. No information
13	What language did the interpreter speak (code for each interpreted interview)	1. The child’s native language
		2. The child’s native language but with a different dialect
		3. The child’s second language (not Swedish)
		4. Another language than the child’s native or second language
		5. No information
14a	Interpreter’s performance (for each interview/interpreter when information is available	1. No remarks
		2. Criticized by the court
		3. Criticized themself
		4. Criticized by another interpreter
		5. Criticized by the defense
		6. Criticized during the preliminary investigation
14b	Describe the criticism	If criticized, describe the criticism
15a	Is the child’s testimony assessed with caution due to interpretation?	1. No
		2. Yes, because an interpreter has been used
		3. Yes, because an interpreter has not been used
15b	Describe court’s assessment	Describe
16	Evidence direct adult crime witness	0 = no, there are no direct crime witness
		1 = yes, someone witnessed the crime
17a	Admission of guilt (from the defendant)	1. Yes
		2. No
		3. Partially
17b	What is admitted?	String variable to describe what is admitted, mainly when the defendant only partially admits to guilt
18a	Evidence behavior (e.g., sad, stomach aches, bed wetting)	0 = No
		1 = yes
18b	Evidence behavior	String variable to describe the behavioral evidence
19a	Evidence medical examination	0 = No medical examination
		1 = medical examination
19b	Medical examination opinion	1. Injuries (indicating crime)
		2. Injuries (uncertain if they are indicating crime)
		3. No injuries
20	Outcome district court	1. Convicted
		2. Dismissed
		3. Partially convicted
21	Penalty outcome	1. Prison
		2. Psychiatric care
		3. Probation
		4. Other (text option)
22	Court of appeal outcome	1. Convicted
		2. Dismissed
		3. Partially convicted
